# Rational Design of Photocontrolled Rectifier Switches in Single-Molecule Junctions Based on Diarylethene

**DOI:** 10.3390/molecules28207158

**Published:** 2023-10-18

**Authors:** Ziye Wu, Peng Cui, Mingsen Deng

**Affiliations:** 1 School of Information, Guizhou University of Finance and Economics, Guiyang 550025, China; zywu@mail.gufe.edu.cn (Z.W.); pcui@mail.gufe.edu.cn (P.C.); 2 Guizhou Provincial Key Laboratory of Computational Nano-Material Science, Guizhou Education University, Guiyang 550018, China

**Keywords:** multifunctional devices, single-molecule junctions, diarylethene, density functional theory

## Abstract

The construction of multifunctional, single-molecule nanocircuits to achieve the miniaturization of active electronic devices is a challenging goal in molecular electronics. In this paper, we present an effective strategy for enhancing the multifunctionality and switching performance of diarylethene-based molecular devices, which exhibit photoswitchable rectification properties. Through a molecular engineering design, we systematically investigate a series of electron donor/acceptor-substituted diarylethene molecules to modulate the electronic properties and investigate the transport behaviors of the molecular junctions using the non-equilibrium Green’s function combined with the density functional theory. Our results demonstrate that the asymmetric configuration, substituted by both the donor and acceptor on the diarylethene molecule, exhibits the highest switching ratio and rectification ratio. Importantly, this rectification function can be switched on/off through the photoisomerization of the diarylethene unit. These modulations in the transport properties of these molecular junctions with different substituents were obtained with molecule-projected self-consistent Hamiltonian and bias-dependent transmission spectra. Furthermore, the current–voltage characteristics of these molecular junctions can be explained by the molecular energy level structure, showing the significance of energy level regulation. These findings have practical implications for constructing high-performance, multifunctional molecular-integrated circuits.

## 1. Introduction

In recent years, the design and construction of high-performance, multifunctional molecular devices have become a significant research focus in molecular electronics [1,2,3,4]. In comparison to semiconductor materials, single-molecule systems are more flexible in their geometry and energy level structures. The energy levels of molecules can be finely tuned, enabling precise control over charge transport at the single-molecule level. In 1974, the concept of a rectifier model with a donor–acceptor system was first proposed by Aviram and Ratner [5]. Until now, numerous functions have been achieved in single-molecule junctions, including rectification [6,7,8], switching [9,10], negative differential resistance [11,12], and a gate effect [13,14], among others. On this basis, extensive experimental and theoretical efforts have been dedicated to enhancing the multifunctionality and performance of molecular devices in order to simplify the circuit design and pave the way for practical applications through the fabrication of multifunctional, single-molecule nanocircuits [15,16,17,18,19,20].

Rectification is a fundamental function of a single-molecule device, often combined with other functions, such as gate regulation, to enhance performance and multifunctionality. However, previous studies have shown limited effectiveness in modulating the rectification ratio of single-molecule devices via a gate [21,22]. Recently, Xin et al. demonstrated that symmetric orbital shifting induced by a gate electric field produced a tunable rectification behavior and a high-performance field effect in an initially symmetric single-molecule field-effect transistor (FET), notably that this effect can be switched on/off by isomerizing the photoresponsive unit [23]. This finding suggests that photoresponsive molecules hold the potential for modulating rectifying behavior. Motivated by this work, we aimed to incorporate a photoresponsive core into a typical molecular rectifier system to achieve a photoswitchable rectification function within a single-molecule junction and investigate its effects on device performance.

**Figure 1:**
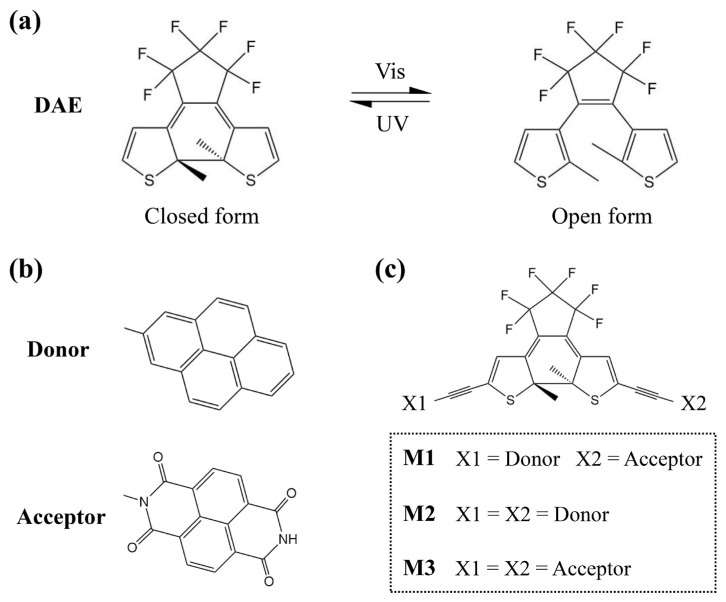
(**a**) Structure and photochemical interconversion of DAE molecule between the ring-closed and ring-open forms. (**b**) Structures of the donor and acceptor groups. (**c**) Molecular structures of M1~M3.

Photoresponsive molecules are commonly utilized as the central components of molecular switch devices, which typically possess two or more stable conformations that can be reversibly converted upon exposure to a specific wavelength of light, resulting in significant variations in conductance [24,25,26]. Diarylethene (DAE) derivatives have been extensively employed in the construction of molecular switch devices due to their excellent thermal stability and fatigue resistance [27,28,29]. The ring-closed and ring-open forms of DAE can rapidly and reversibly interconvert via electrocyclization under ultraviolet/visible (UV/Vis) light irradiation (Figure 1a). Following the successful fabrication of reversible molecular junctions using phenylene-based DAE by Kudernac et al. [30], subsequent efforts have led to the development of single-molecule devices featuring high performances based on DAE cores [31,32]. Recently, Meng et al. reported a dual-gated single-molecule FET based on a ruthenium–DAE complex that achieved a maximum on/off ratio exceeding 10^3^ [33], thereby reaffirming the remarkable switching performance and stable reversible switching ability exhibited by DAE molecules.

In this work, we designed a donor–DAE–acceptor system containing a pyrene group and a 1,4,5,8-naphthalenetetracarboxylic diimide group at the two mirror-asymmetric positions of the thiophene rings in DAE (see Figure 1b,c). For comparison, the symmetrical donor–DAE–donor and acceptor–DAE–acceptor systems were also studied. We first confirm that the three molecules have distinct charge distributions and energy level structures based on density functional theory (DFT) calculations. Then, their electron transport properties are examined using the non-equilibrium Green’s function (NEGF) in combination with DFT. The results show that the asymmetric configuration exhibits the highest switching ratio and rectification ratio. Importantly, the rectifying function caused by the asymmetric configuration can be switched on/off through the photoisomerization of the DAE core.

## 2. Results and Discussion

### 2.1. Electronic Properties of Single Molecules

Based on a molecular engineering design, an asymmetric photoswitchable system M1 centered on DAE was obtained by incorporating both donor and acceptor substituents (Figure 1). Here, we select pyrene and 1,4,5,8-naphthalenetetracarboxylic diimide as donor and acceptor substituents, respectively, due to their similar frameworks. Symmetrically substituted molecules M2 and M3 are also investigated for comparison. The reversible photoisomerization between the conjugated ring-closed isomer with high conductance and the unconjugated ring-open isomer with low conductance forms the basis of DAE as a reliable molecular switch. To examine the influence of donor and acceptor groups on the photochemical properties of the molecules, we simulated the absorption spectra of DAE as well as M1~M3 (Figure S1, Supplementary Materials). The absorption maxima for the ring-closed DAE and M1~M3 are observed at 454 nm, 537 nm, 547 nm and 527 nm, while those for ring-open isomers occur at 273 nm, 343 nm, 328 nm and 365 nm, respectively. In both the gas phase and the solvent, M1~M3 exhibit red shifts in their absorption spectra compared to the prototype DAE molecule (Figure S1). However, the ring-closed and ring-open isomers still respond to visible and ultraviolet light accordingly.

**Figure 2:**
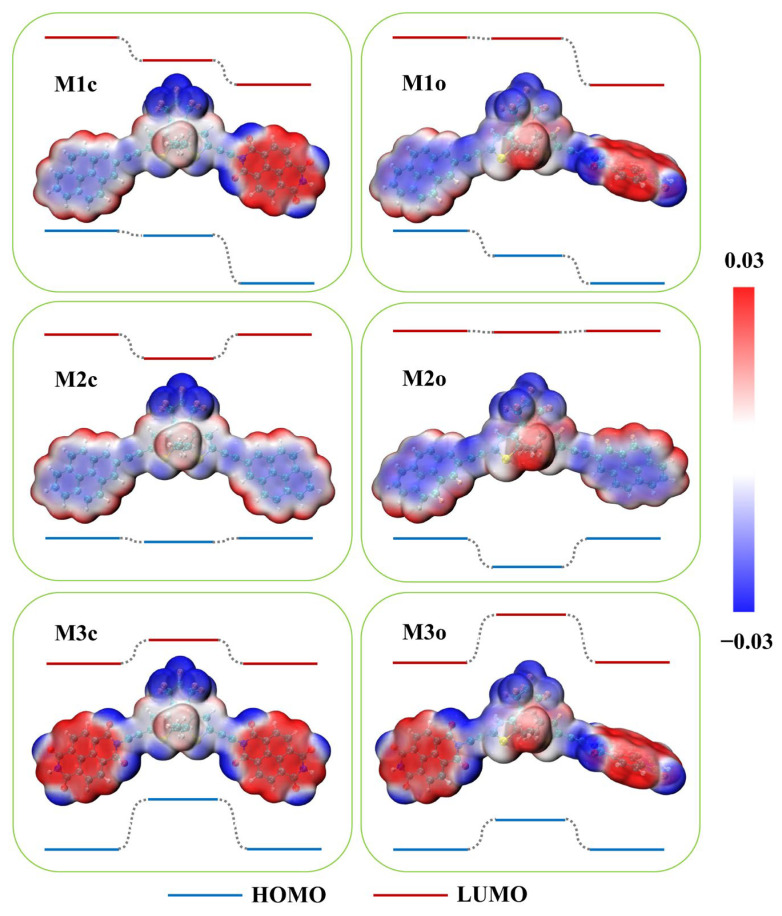
Calculated electrostatic potential mapping of the ring-closed and ring-open forms of single-molecule M1~M3, showing the relative energy level diagram for each part.

For convenience, the ring-closed/ring-open form is marked as c/o. The calculated electrostatic potential reveals distinct charge distributions between the donor and acceptor (Figure 2). The fused ring of the donor exhibits a negative charge due to delocalized π-bonds, while that of the acceptor is positively charged owing to its four electron-withdrawing oxygen atoms. Although molecular energy levels are commonly used to explain intramolecular electronic behavior, energy levels of the whole molecule provide limited information. For example, the ring-closed isomers of M1~M3 have similar excitation energies (with differences less than 0.1 eV) and energy gaps (Table S1, and the results in the solvent are shown in Table S2), which cannot adequately account for variations in electronic properties among them. To determine the relative energy levels of each component in M1~M3, we calculated the energy levels of the isolated neutral systems for DAE, the donor and the acceptor (Table S3), and confirmed that this treatment is qualitatively reasonable. As shown in Figure 2, the lowest molecular orbital (LUMO) of M1c exhibits a stepped structure indicative of a directed charge transfer. The calculated total number of transferred electrons from the five lowest excited states confirm that there are 0.353 electrons transferred from the donor (X1) to the switch and 0.958 electrons transferred from the switch to the acceptor (X2), respectively (see Table 1, detailed information on each excited state can be found in Table S5). In M1o, the elevation of the LUMO energy in the DAE part breaks the stepped structure, resulting in a negligible number of electron transfers from the donor to the switch with just 0.033 electrons (Table 1). In contrast, M2c’s LUMO displays a concave structure (Figure 2), resulting in the transfer of 0.279 and 0.273 electrons from the two donors (X1 and X2) to the switch, respectively (Table 1). However, minimal electron transfers occur in M2o (<0.03 electrons, see Table 1) due to the closed LUMO energies between the DAE and donor moiety (Figure 2). In M3c and M3o, the electrons transfer from the switch to the acceptors, X1 and X2 (Table 1), because of the convex structure of LUMO (Figure 2).

### 2.2. Current–Voltage (I–V) Characteristics

**Figure 3:**
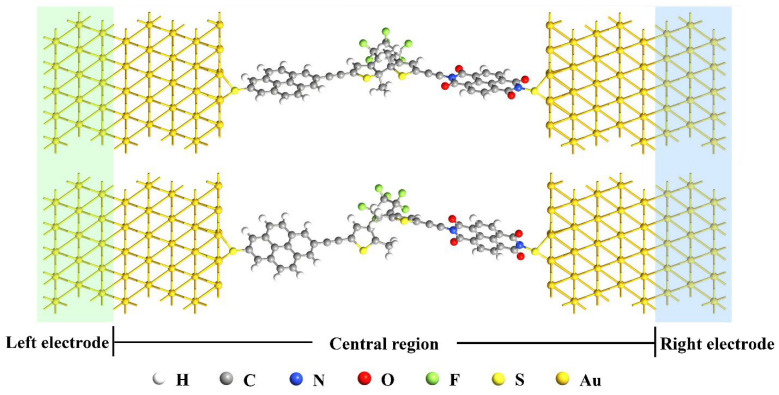
Geometric structures of the single-molecule junctions for closed form (up) and open form (down) with Au(111) electrodes.

We have demonstrated that the charge distributions and energy level structures of M1~M3 can be modulated by varying the donor/acceptor substituents on DAE, which undoubtedly exert a significant influence on the electron transport characteristics. To simulate an actual device, we constructed single-molecule junctions, as shown in Figure 3 (taking M1 as an example), where both the ring-closed and ring-open isomers of M1~M3 are connected to two Au(111) electrodes via a sulfur atom. Based on the well-optimized configurations of these junctions, subsequent electronic transport calculations were performed.

**Figure 4:**
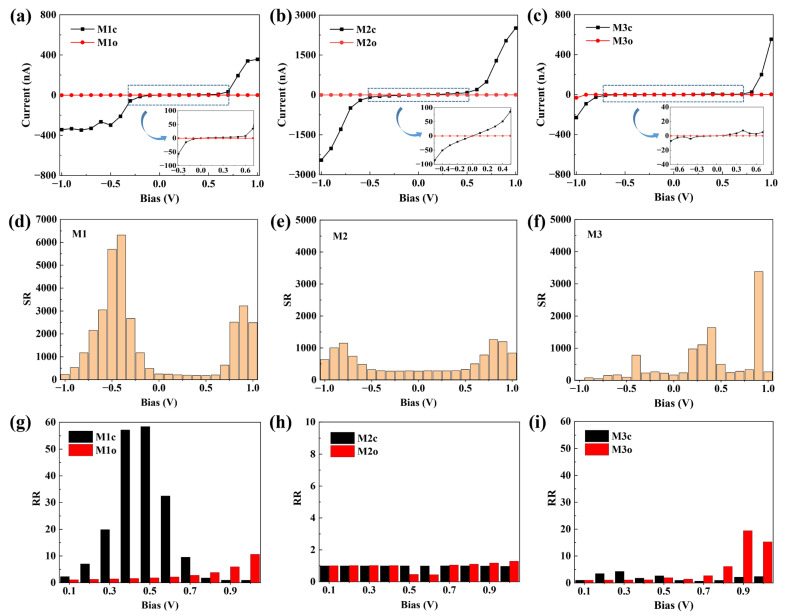
I–V curve (**a**–**c**), current switching ratio (**d**–**f**) and current rectification ratio (**g**–**i**) of molecular junctions M1~M3.

The calculated I–V characteristics of the molecular junctions within a bias voltage range of [−1.0, 1.0 V] are presented in Figure 4a–c. Notably, the current flowing through the ring-closed configurations is remarkably higher than that through the ring-open counterparts. As shown in Table 2, the zero-bias transmission for the closed form is at least two orders of magnitude lager than that for the corresponding open form. These results unequivocally demonstrate that all of the molecular junctions exhibit switching effects. In order to quantitatively describe the switching performance, the current switching ratio is defined as *SR*(*V*) = *I*_closed_(*V*)/*I*_open_(*V*). In particular, the switching ratio at zero bias is calculated based on the transmission (i.e., *T*_0_(closed)/*T*_0_(open)). The maximum switching ratios of the M1~M3 junctions are 6319, 1261 and 3381, respectively (Table 2). It should be emphasized that M1 exhibits an impressive switching ratio reaching up to 10^3^ within a wide negative bias voltage range of [−0.8, −0.2 V] (Figure 4d), and M2 ensures a stable switching ratio exceeding 200 throughout the entire bias range [−1.0, 1.0 V] (Figure 4e). However, M3 exhibits an unstable switching ratio that varies from 7 to 3381 (Figure 4f). This implies that both M1 and M2 can be utilized as molecular devices with reliable switching performances, and in particular, the switching ratio exhibited by M1 when an appropriate bias is applied is exceptional.

Additionally, due to its asymmetric configuration, M1c exhibits pronounced rectification characteristics (Figure 4a). Here, we defined the current rectification ratio as *RR*(*V*) = |*I*(−*V*)/*I*(+*V*)|. The maximum rectification ratio of the M1c junction reaches 58.4, whereas this value remains below 5 in the case of the M2c and M3c junctions (Figure 4g–i). Even in the presence of asymmetry in M1o, the maximum rectification ratio is only 10.6 (Table 2). In particular, at a bias of 0.5 V, the rectification ratios for M1c and M1o are observed to be 58.4 and 1.8 respectively. This indicates that M1 can effectively achieve the conversion to rectifying behavior during light-driven processes involving transformation between ring-closed and ring-open forms, which means that the photoswitchable rectification function is realized in the same single-molecule junction.

### 2.3. Explanations of I−V Characteristics

**Figure 5:**
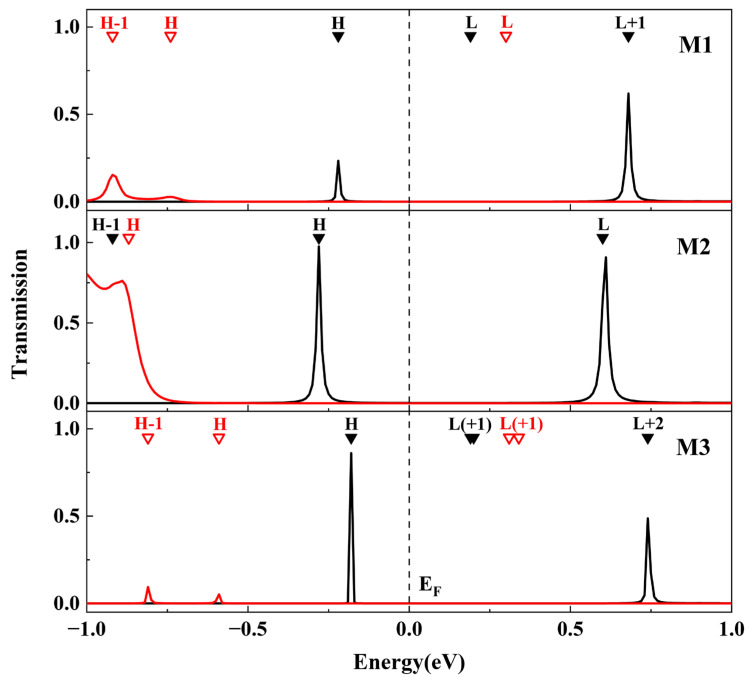
Transmission spectra of the closed form (black line) and open form (red line) for molecular junction M1~M3 at zero bias. The *E*_F_ is set at zero in the energy scale. The solid (closed form) and hollow (open form) down-pointing triangles denote the MPSH eigenvalues of each molecule. H and L denote HOMO and LUMO, respectively.

The zero-bias transmission spectra of the M1~M3 junctions were calculated and are presented in Figure 5. It is evident that the transmission spectra exhibit distinct differences between the ring-closed and ring-open isomers. Specifically, there are two sharp transmission peaks within the energy range [−0.5, 1.0 eV] for each closed configuration, whereas the peaks for the open configuration are located far from the Fermi level (*E*_F_) and have a lower energy (<−0.5 eV). This discrepancy in zero-bias transmission between the ring-closed and ring-open isomers can be attributed to this main cause (Table 2).

**Figure 6:**
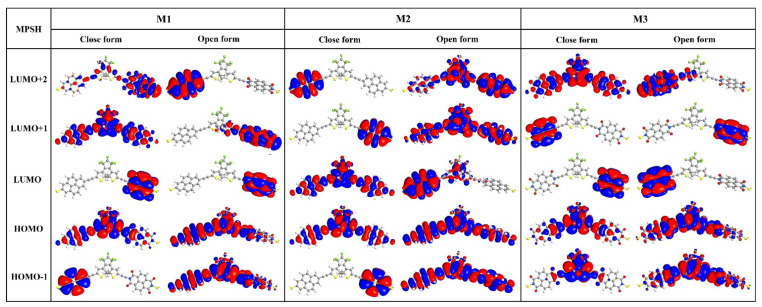
Spatial distribution of the frontier molecular orbitals in molecular junctions M1~M3 at zero bias.

In order to elucidate the origin of these transmission peaks, we employed the molecule-projected self-consistent Hamiltonian (MPSH) technique to calculate both the eigenvalues and the spatial distribution of frontier orbitals, as labeled in Figure 5 and shown in Figure 6. In this analysis, the self-consistent Hamiltonian of the entire junction is projected onto the molecule part, including anchoring atoms, so as to account for the impact of the electrodes. As shown in Figure 5, the eigenvalues corresponding to HOMO in each junction align well with positions of the respective transmission peak closest to *E*_F_, indicating that HOMO serves as a primary electronic transport channel. Furthermore, the asymmetric distribution in the HOMO of M1c reflects varying strengths of interaction with two electrodes because the anchoring atom attached to the donor exhibits a greater orbital distribution compared to that attached to the acceptor (Figure 6). No discernible transmission near LUMO is observed for M1 (both M1c and M1o, see Figure 5), primarily due to its localization on the acceptor moiety (Figure 6). Although LUMO+1 in M1c is a delocalized state, its energy level (0.68 eV) lies further away from *E*_F_ than HOMO (−0.22 eV), thereby limiting its contribution towards electronic transport. A similar situation exists for M3c.

**Figure 7:**
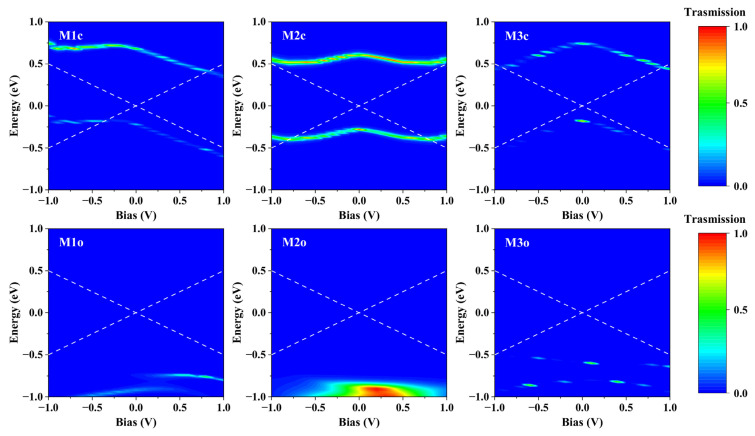
Bias-dependent transmission spectra of molecular junctions M1~M3. The white dashed lines in each subgraph represent the bias window.

To further understand the transport behavior with the increasing bias in the molecular junctions M1~M3, we calculated the transmission spectra at varying bias voltages, as shown in Figure 7. The current flowing through the molecular junction depends on the integral area of the transmission spectrum within the bias window. In contrast to the closed configuration, all transmission spectra of the open forms are significantly far from the bias window and can be considered negligible, resulting in switching effects in M1~M3. Moreover, the transmission peak at the HOMO of M1c hardly moves when the bias increases negatively and enters the bias window after −0.3 V, but it moves away from the bias window when the bias increases positively until the transmission peak at LUMO+1 enters the bias window at approximately 0.8 V. Consequently, there is a difference in the bidirectional currents for M1c, and it exhibits a rectification characteristic.

**Figure 8:**
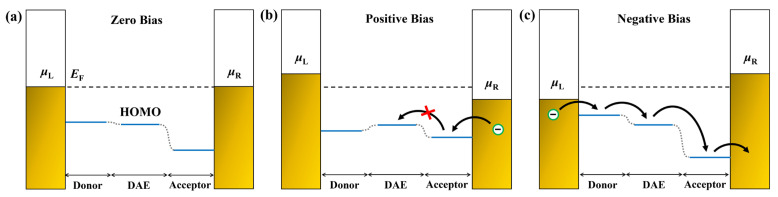
Schematic demonstration of the charge transport of M1c junction under zero bias (**a**), positive bias (**b**) and negative bias (**c**), respectively. The blue lines represent the relative energy level of HOMO for each part.

More simply, the differences in the I–V characteristics among these molecular devices can be effectively elucidated by considering the relative energy level structure of the molecules. Here, we focus solely on discussing the I–V characteristics of ring-closed configurations, because all ring-open configurations exhibit no obvious increase in the current within the bias range of [−1.0, 1.0 V] (Figure 4a–c). As depicted in Figure 8, when a bias voltage is applied to the left or right electrode, it causes a shift in the energy level for the donor or acceptor moiety in contact with it. The relative HOMO energy levels in M1c are illustrated in Figure 8b under the positive bias condition, where electrons are injected into the acceptor from the right electrode with a lower potential. However, unless a higher bias is applied to elevate the HOMO of the acceptor above that of the DAE, it becomes challenging for electrons residing on the acceptor to overcome their energy barrier and transfer to the DAE. Conversely, the relative HOMO levels of M1c under the negative bias condition exhibit a step-like structure as shown in Figure 8c, which facilitates an easy electron transfer from the donor to the DAE and subsequently to the acceptor along descending energy levels. Consequently, a positive bias necessitates a higher turn-on voltage for activating charge transport compared to negative bias conditions (as shown in Figure 4a with turn-on voltages are 0.6 V and −0.2 V, respectively). Similarly, M2c requires only a small turn-on voltage due to the minimal energy difference between the donor and DAE (Figure 1), resulting in continuous current increment values (Figure 4b). In contrast, M3c demands a high turn-on voltage (~±0.8 V, see Figure 4c) to overcome the significant energy barrier between the acceptor and DAE (Figure 1).

## 3. Models and Methods

The molecular structures in the ground state of M1~M3 with ring-closed and ring-open forms were first optimized with the Gaussian09 program [34]. The long-range-corrected hybrid density functional CAM-B3LYP [35] was applied with the 6-311G(d,p) basis set. Time-dependent density functional theory (TD-DFT) calculations at the same theoretical level were carried out to simulate the electronic excited states. All of the above calculations were performed in the gas phase, and the solvent effect was also taken into account by using the polarizable continuum model (PCM) [36] for comparison. Then, electrostatic potential [37] and interfragment charge transfer (IFCT) [38] analyses was performed using the Multiwfn 3.8(dev) program [39].

The single-molecule junction models are illustrated in Figure 3, which can be divided into three components: the left electrode, the central region and the right electrode. The central region consists of a single molecule with a ring-closed or ring-open form, which is bridged to the Au(111) surface by a sulfur atom at each end. Each Au(111) electrode is represented by a 5 × 5 supercell with periodic boundary conditions. Geometry optimizations and the determination of the electronic transport properties of the molecular junctions were carried out using the DFT and NEGF methods implemented in the QuatumATK software [40]. The exchange and correlation energies of valence electrons were computed using the Perdew–Burke–Ernzerhof (PBE) functional [41] within the framework of generalized gradient approximation (GGA), and the core electrons are described by norm-conserving Troullier–Martins pseudopotentials [42]. A single-zeta plus polarization (SZP) basis set was employed for Au atoms, and a double-zeta plus polarization (DZP) basis set was used for other atoms. The mesh cut-off was set to 300 Rydberg, and for the Brillouin-zone *k*-sampling, we employed a 2 × 2 × 130 grid. The convergence criterion for the Hamiltonian during the self-consistent calculations was set to be a 1.0 × 10^−5^ Hartree.

The tunneling currents through the molecular junctions can be calculated using the Landauer–Büttiker formula [43]:

(1) $$I\left(V\right)=\frac{2e}{h}\int T\left(E,V\right)\left[f\left(E-\mu_{L}\right)-f\left(E-\mu_{R}\right)\right]dE$$

Here, *V* is the bias voltage applied to the molecular junctions, *T*(*E*, *V*) is the bias-dependent transmission coefficient, $f\left(E-\mu_{L}\right)$/$f\left(E-\mu_{R}\right)$ is the Fermi–Dirac distribution functions of the left/right electrodes, and $\mu_{L}$/$\mu_{R}$ is the chemical potential of the left/right electrodes.

## 4. Conclusions

In summary, we propose a design strategy for achieving a high-performance, photocontrolled rectifier based on a DAE molecular switch. The electronic transport properties of single-molecule junctions, where a DAE molecule is substituted with electron donor/acceptor groups and connected to two gold electrodes, were investigated using the NEGF and DFT methods. The influence of substituents on transport behavior and its underlying mechanism were discussed. Compared to symmetrically substituted molecules, the asymmetric molecule with both donor and acceptor groups exhibits a superior switching performance, with a switching ratio above 10^3^ over a wide bias range. Additionally, its rectification function can be switched on/off through photoisomerization. This rational design offers a general and effective strategy to enhance switching performance and enable the integration of multiple functions, which is of great importance for the study of molecular properties and the construction of high-performance devices. Furthermore, the differences in transport properties among these molecular devices can be explained by their relative energy levels, highlighting the importance of adjusting energy level structures for designing single-molecule devices with specific functions. 

## Figures and Tables

**Table 1:** Total transferred electrons between fragments *^a^* in M1~M3.

	Total Transferred Electrons *^a^* (e^−^)		
Molecule	X1→Switch *^b^*	Switch→X2 *^b^*	X1→X2 *^b^*
M1c	0.353	0.958	1.032
M1o	0.033	1.505	1.338
M2c	0.279	−0.273	−0.000
M2o	0.020	−0.025	−0.002
M3c	−0.973	0.973	−0.001
M3o	−0.807	1.168	0.005

*^a^* Total transferred electrons are the sum of the transferred electrons in the five lowest excited states. The negative sign means reverse electron transfer. *^b^* Each molecule is divided into three fragments, where X1 and X2 are the donor or acceptor groups (see Figure 1c), and the switch includes the rest of the parts, except X1 and X2.

**Table 2:** Zero-bias transmission (*T*_0_), maximum switching ratio (*SR_max_*) and maximum rectification ratio (*RR_max_*) of molecular junctions M1~M3.

	M1	M2	M3
*T*_0_ (closed form)	1.68 × 10^−4^	1.26 × 10^−3^	2.03 × 10^−5^
*T*_0_ (open form)	6.76 × 10^−7^	4.60 × 10^−6^	1.22 × 10^−7^
*SR_max_*	6319	1261	3381
*RR_max_* (closed form)	58.4	1.0	4.3
*RR_max_* (open form)	10.6	1.3	19.4

## Data Availability

Data are available upon request.

## Supplementary Materials

The following supporting information can be downloaded at: https://www.mdpi.com/article/10.3390/molecules28207158/s1. Figure S1: Calculated UV/Vis spectra of DAE, M1, M2 and M3; Table S1: Molecular orbital energies of molecule M1~M3 in gas phase; Table S2: Molecular orbital energies of molecule M1~M3 in solvent; Table S3: Molecular orbital energies of donor, DAE and acceptor in gas phase; Table S4: Molecular orbital energies of donor, DAE and acceptor in solvent; Table S5: Transferred electrons between fragments in molecule M1~M3 in gas phase; Table S6: Transferred electrons between fragments in molecule M1~M3 in solvent; Table S7: MPSH eigenvalues of each molecule in single-molecule junctions.
