# Supplementary material for: Rational Design of Photocontrolled Rectifier Switches in Single-Molecule Junctions Based on Diarylethene
Source: Molecules. 2023 Oct 18;28(20):7158. doi: 10.3390/molecules28207158 (PMC10609135; doi:10.3390/molecules28207158)
Supplement: Supplementary file 1 [file molecules-28-07158-s001.zip › molecules-2651366-supplementary.pdf]

Supplementary Materials

Rational Design of Photocontrolled Rectifier Switches in Single-Molecule Junctions Based on Diarylethene

Ziye Wu[1], Peng Cui[1] and Mingsen Deng[1,2,*]

[1] *School of Information, Guizhou University of Finance and Economics, Guiyang 550025, China*

[2] *Guizhou Provincial Key Laboratory of Computational Nano-Material Science, Guizhou Education University, Guiyang 550018, China*

* Correspondence: deng@gznc.edu.cn

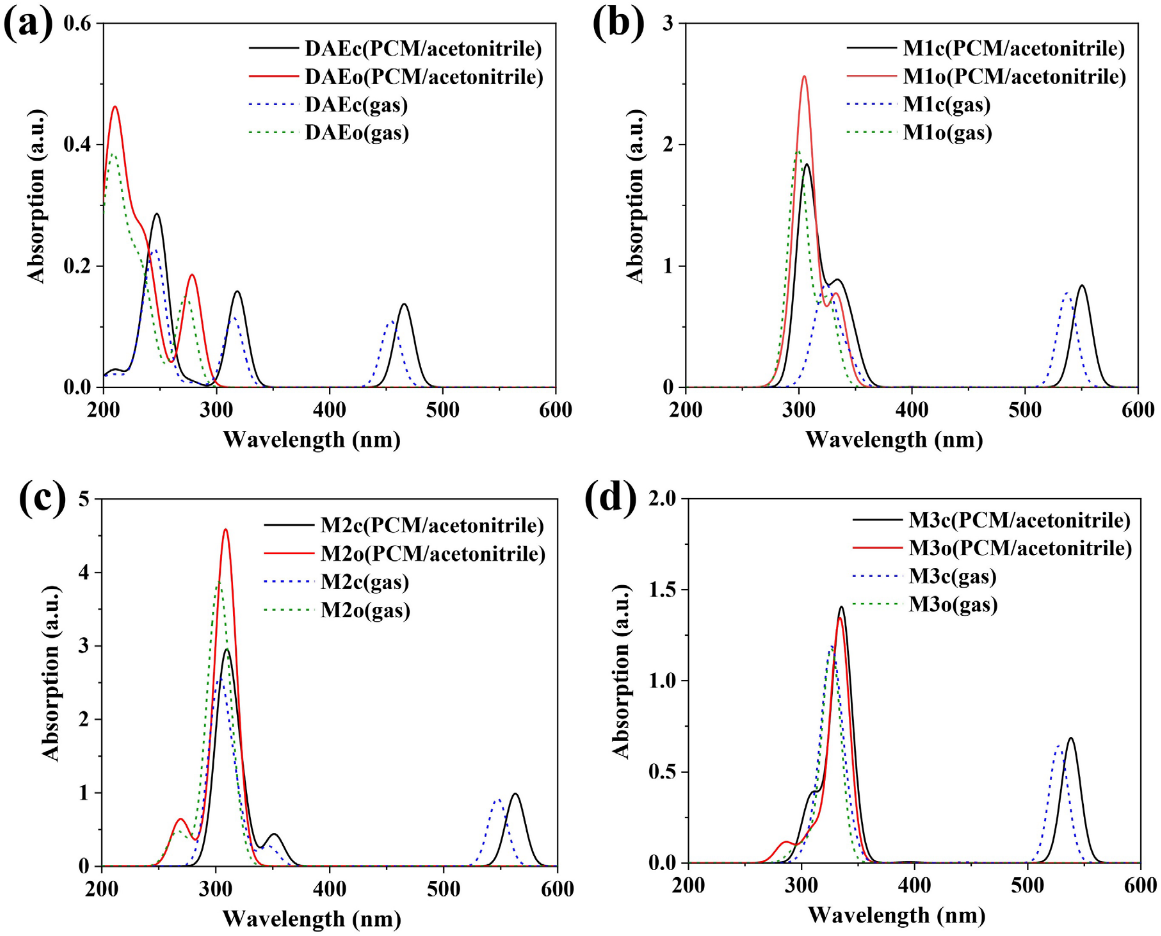

Figure S1. Calculated UV/Vis spectra of DAE (a), M1 (b), M2 (c) and M3 (d) respectively, performed using Gaussian 09 software at theoretical level TD-DFT/CAM-B3LYP/6-311G(d,p)/PCM(acetonitrile) and TD-DFT/CAM-B3LYP/6-311G(d,p)/gas.

Table S1. Molecular orbital energies of molecule M1~M3 calculated at theoretical level TD-DFT/CAM-B3LYP/6-311G(d,p)/gas using Gaussian 09 software.

Energy of molecular orbital (eV)	M1		M2		M3	
	Closed form	Open form	Closed form	Open form	Closed form	Open form
LUMO+1	-2.09	-1.03	-0.92	-0.80	-2.86	-2.76
LUMO	-2.86	-2.76	-2.10	-0.89	-2.87	-2.77
HOMO	-6.63	-7.01	-6.57	-7.01	-6.69	-7.66
HOMO-1	-7.12	-7.19	-7.11	-7.02	-8.06	-8.01
Gap	3.77	4.25	4.47	6.12	3.82	4.89

Table S2. Molecular orbital energies of molecule M1~M3 calculated at theoretical level TD-DFT/CAM-B3LYP/6-311G(d,p)/PCM(acetonitrile) using Gaussian 09 software.

Energy of molecular orbital (eV)	M1		M2		M3	
	Closed form	Open form	Closed form	Open form	Closed form	Open form
LUMO+1	-2.17	-0.88	-0.98	-0.85	-2.56	-2.53
LUMO	-2.57	-2.53	-2.21	-0.90	-2.57	-2.53
HOMO	-6.71	-7.06	-6.66	-7.10	-6.77	-7.61
HOMO-1	-7.01	-7.23	-7.10	-7.10	-8.14	-7.98
Gap	4.14	4.53	4.45	6.15	4.20	5.08

Table S3. Molecular orbital energies of donor, DAE and acceptor calculated at theoretical level TD-DFT/CAM-B3LYP/6-311G(d,p)/gas using Gaussian 09 software.

Energy of molecular orbital (eV)	Donor	DAE		Acceptor
		Closed form	Open form	
LUMO+1	0.26	0.42	0.39	-0.66
LUMO	-0.61	-1.66	-0.66	-2.62
HOMO	-6.79	-6.96	-7.99	-8.72
HOMO-1	-7.73	-8.19	-8.42	-9.45
Gap	6.18	5.30	7.33	6.10

Table S4. Molecular orbital energies of donor, DAE and acceptor calculated at theoretical level TD-DFT/CAM-B3LYP/6-311G(d,p)/PCM(acetonitrile) using Gaussian 09 software.

Energy of molecular orbital (eV)	Donor	DAE		Acceptor
		Closed form	Open form	
LUMO+1	0.10	0.54	0.52	-0.43
LUMO	-0.75	-1.55	-0.61	-2.43
HOMO	-6.93	-6.82	-7.87	-8.49
HOMO-1	-7.89	-8.07	-8.27	-9.16
Gap	6.18	5.27	7.26	6.06

Table S5. Transferred electrons between fragments in molecule M1~M3 analyzed by Multiwfn 3.8 (dev) program based on the TD-DFT calculations at theoretical level TD-DFT/CAM-B3LYP/6-311G(d,p)/gas.

Molecule	Excited state[a]	Transferred electrons (e^-)		
		X1→Switch	Switch→X2	X1→X2
M1c	S_1	-0.00972	0.01550	0.00039
	S_2	0.00000	0.92577	0.02587
	S_3	0.36276	0.00005	0.00533
	S_4	-0.00012	0.01686	0.00077
	S_5	0.00000	0.00007	0.99992
	**Sum**	**0.35292**	**0.95825**	**1.03228**
M1o	S_1	0.00000	0.86831	0.04157
	S_2	0.03335	0.00000	0.00000
	S_3	0.00000	0.00000	1.00000
	S_4	0.00000	-0.00018	-0.00033
	S_5	0.00000	0.63713	0.29657
	**Sum**	**0.03335**	**1.50526**	**1.33781**
M2c	S_1	-0.01131	0.01211	0.00003
	S_2	0.35834	0.00004	0.00663
	S_3	0.00276	0.00433	0.00031
	S_4	-0.00021	-0.36051	-0.00679
	S_5	-0.07020	0.07123	-0.00066
	**Sum**	**0.27938**	**-0.27280**	**-0.00048**
M2o	S_1	0.03023	-0.00005	-0.00047
	S_2	0.00000	-0.03188	0.00000
	S_3	-0.00746	0.00585	-0.00028
	S_4	-0.00082	0.00081	-0.00030
	S_5	-0.00174	0.00072	-0.00141
	**Sum**	**0.02021**	**-0.02455**	**-0.00246**
M3c	S_1	-0.01509	0.01583	0.00001
	S_2	-0.00651	0.93091	0.01006
	S_3	-0.93192	0.00651	-0.01122
	S_4	-0.02054	0.02083	0.00002
	S_5	0.00108	-0.00116	-0.00016
	**Sum**	**-0.97298**	**0.97292**	**-0.00129**
M3o	S_1	-0.80717	0.00000	-0.00032
	S_2	0.00000	0.90863	0.00242
	S_3	-0.00013	0.00009	0.00000
	S_4	0.00001	-0.00001	-0.00022
	S_5	0.00000	0.25944	0.00284
	**Sum**	**-0.80729**	**1.16815**	**0.00472**

[a] S_1~S_5 denote the first to fifth excited states, respectively.

Table S6. Transferred electrons between fragments in molecule M1~M3 analyzed by Multiwfn 3.8 (dev) program based on the TD-DFT calculations at theoretical level TD-DFT/CAM-B3LYP/6-311G(d,p)/PCM(acetonitrile).

Molecule	Excited state[a]	Transferred electrons (e^-)		
		X1→Switch	Switch→X2	X1→X2
M1c	S_1	-0.00931	0.01081	0. 00025
	S_2	0.00000	0.91113	0.02490
	S_3	0.44332	-0.00019	0.00504
	S_4	0.00876	0.01149	0.00099
	S_5	0.00001	-0.00109	0.00023
	**Sum**	**0.44278**	**0.93215**	**0.03116**
M1o	S_1	0.00000	-0.00088	0.00021
	S_2	0.04371	0.00000	0.00000
	S_3	0.00000	0.79770	0.02454
	S_4	-0.01689	0.00009	0.00024
	S_5	0.00000	0.13345	0.02253
	**Sum**	**0.02682**	**0.93036**	**0.04752**
M2c	S_1	-0.00744	0.00910	0.00006
	S_2	0.45167	0.00002	0.00758
	S_3	-0.00004	-0.44558	-0.00861
	S_4	0.00805	-0.00526	0.00014
	S_5	-0.04517	0.04777	0.00000
	**Sum**	**0.40707**	**-0.39395**	**-0.00083**
M2o	S_1	0.04298	0.00054	0.00004
	S_2	0.00004	0.00008	-0.04629
	S_3	-0.01611	0.01415	0.00051
	S_4	0.00296	0.00409	0.00131
	S_5	-0.00655	0.00663	0.00118
	**Sum**	**0.02332**	**0.02549**	**-0.04325**
M3c	S_1	-0.00943	0.01022	0.00001
	S_2	-0.00002	0.92376	0.01074
	S_3	-0.92486	0.00002	-0.01117
	S_4	-0.01366	0.01431	0.00008
	S_5	0.00014	-0.00015	-0.00018
	**Sum**	**-0.94783**	**0.94816**	**-0.00052**
M3o	S_1	-0.76533	0.00000	-0.00105
	S_2	-0.00045	0.00024	0.00000
	S_3	0.00002	-0.00005	-0.00034
	S_4	-0.00018	0.77807	0.00105
	S_5	0.00000	0.18201	0.00073
	**Sum**	**-0.76594**	**0.96027**	**0.00039**

[a] S_1~S_5 denote the first to fifth excited states, respectively.

Table S7. Molecule-projected self-consistent Hamiltonian (MPSH) eigenvalues of each molecule in single-molecule junctions simulated by QuatumATK software.

MPSH eigenvalues (eV)	M1		M2		M3	
	Closed form	Open form	Closed form	Open form	Closed form	Open form
LUMO+2	1.44	1.67	1.48	1.74	0.74	1.36
LUMO+1	0.68	1.41	1.47	1.61	0.20	0.34
LUMO	0.19	0.30	0.60	1.60	0.19	0.31
HOMO	-0.22	-0.74	-0.28	-0.87	-0.18	-0.59
HOMO-1	-1.15	-0.92	-0.92	-1.03	-1.48	-0.81
Gap	0.41	1.04	0.88	2.47	0.37	0.90
